# Trends in Myocardial Infarction risk by HIV status in two U.S. healthcare systems

**DOI:** 10.1371/journal.pone.0325773

**Published:** 2025-06-23

**Authors:** Alexandra N. Lea, Asya Lyass, Leo B. Hurley, Rachel Q. Ehrbar, Taylor F. Mahoney, Leila H. Borowsky, Wei He, Jorge Plutzky, Virginia A. Triant, Michael J. Silverberg

**Affiliations:** 1 Division of Research, Kaiser Permanente Northern California, Pleasanton, California, United States of America; 2 Department of Mathematics and Statistics, Boston University, Boston, Massachusetts, United States of America; 3 Department of Biostatistics, Boston University School of Public Health, Boston, Massachusetts, United States of America; 4 Division of General Internal Medicine, Massachusetts General Hospital, Boston, Massachusetts, United States of America; 5 Division of Cardiovascular Medicine, Brigham and Women’s Hospital, Boston Massachusetts, United States of America; 6 Division of Infectious Diseases, Massachusetts General Hospital, Boston, Massachusetts, United States of America; 7 Mongan Institute, Massachusetts General Hospital, Boston, Massachusetts, United States of America; Taipei Medical University, TAIWAN

## Abstract

People with HIV have a higher risk of myocardial infarction (MI) compared with people without HIV. Real world evidence on whether the increased burden of MI in people with HIV is sustained over time is essential for understanding cardiovascular disease (CVD) patterns, optimizing management, and identifying unmet needs. To help fill this gap, we completed a cohort study of people with HIV and propensity matched people without HIV in two distinct US-based integrated healthcare systems (Kaiser Permanente Northern California and Mass General Brigham). Electronic health records were assessed for association of HIV status on MI risk in two calendar eras defined by baseline year: 2005–2009 and 2010–2017, with maximum 5-year follow-up, through 2020. Cox proportional hazards models were used to obtain adjusted hazard ratios (HR) for HIV status on MI risk overall and by cohort. Adjusted models controlled for demographics and traditional CVD risk factors. We included 9,401 people with HIV (78 with MI) and 29,418 people without HIV (204 with MI). In adjusted models, the HR for MI in people with HIV was 1.10 (95% CI, 0.76, 1.60) for years 2005–2009 and 1.66 (95% CI, 1.15, 2.39) for years 2010–2017 compared with people without HIV, with a corresponding P-interaction between HIV status and calendar era of 0.12. Differences were largely due to decreases in MI risk for people without HIV. The magnitude of HRs by calendar era was consistent across models stratified by cohort. Results showed an increased MI risk persisted among people with HIV in recent years relative to people without HIV, even as CVD rates are decreasing in the general population. In light of known HIV-specific CVD risk factors, continued surveillance for MIs is merited.

## Introduction

While cardiovascular disease (CVD) remains the leading cause of morbidity and mortality in the United States [1], people with HIV have a two-fold greater risk of myocardial infarction (MI) compared with people without HIV [2]. Contributing factors to increased MI risk associated with HIV include the greater prevalence of traditional CVD risk factors [3], possible adverse effects of certain antiretroviral therapy (ART) medications [4–10], and mechanisms of atherosclerotic complications directly related to HIV, including HIV-mediated inflammation and immune dysregulation, which may persist even with successful ART [11–13]. HIV infection is associated with inflammation through chronic immune system activation, including by opportunistic infections and persistent virus even in people with HIV whose virus is well-controlled [14–16]. Chronic inflammation of the metabolic system is also associated with HIV infection and treatment with ART [17], which can contribute to weight gain [18,19] and lipid dysregulation [20]. As a result, real world data on MI rates by HIV status in large, diverse cohorts are essential for understanding CVD patterns, optimizing management, and identifying unmet needs in this population.

The objective of this study was to quantify changes in MI incidence rates over time by comparing people with HIV and people without HIV identified from two large and geographically disparate healthcare systems, with participants selected to have similar baseline risks of MI.

## Materials and methods

### Study design, setting and population

We conducted a cohort study among adult (aged ≥18 years) people with HIV and people without HIV at Kaiser Permanente Northern California (KPNC) and at Mass General Brigham (MGB), an integrated healthcare system in Massachusetts. KPNC has 4.5 million current members across Northern California, with ~30,000 cumulative members with HIV, while MGB, whose main institutions are Brigham and Women’s and Massachusetts General Hospital, serves ~1.5 million individuals, with ~7,000 cumulative people with HIV served.

Adult (age ≥ 18 years old) people with HIV and people without HIV were eligible if they had active health plan membership (KPNC) or longitudinal follow-up (MGB) between January 1, 2005 and December 31, 2017 and no prior diagnosis of CVD.

People without HIV were propensity score matched 3:1 in KPNC and 4:1 in MGB, using baseline demographics (age, sex at birth, race/ethnicity, year of baseline) and baseline Framingham risk score components (total cholesterol, high-density lipoprotein [HDL], diabetes, systolic blood pressure [SBP], hypertension treatment, and smoking status).

All study procedures were reviewed and waivers of informed consent were approved by the KPNC and MGB Institutional Review Boards. Data was accessed between August 1, 2018 and June 30, 2021 for both KPNC and MGB and only data managers (authors AL and LBH) had access to identifiable information for individual participants during the data collection process.

### Study period

Baseline was defined as first year that Framingham risk score components were measured, anchored at date of lipids measurement. Study follow-up occurred from January 1, 2006 to December 31, 2020. Follow-up ended at earliest of: MI, death, loss-to-follow-up, 5 years after baseline or end of follow-up (December 31, 2020).

### Data sources

Sociodemographic and clinical data were obtained from patient electronic health records (EHR) at both sites, which also includes laboratory and pharmacy data. In KPNC, HIV status was ascertained through membership in the KPNC HIV Registry. MGB identified people with HIV through a validated algorithm [21].

### Outcomes & Exposures

Outcome was incident MI (defined as ICD-9 410.xx or ICD-10 I21). All events were adjudicated at MGB and a validated case definition was used at KPNC, with a ≥ 95% positive predictive value for myocardial infarction [22]. Primary exposures were HIV status and baseline calendar era (2005–2009 and 2010–2017).

### Covariates

Components of the Framingham Risk Score for Hard Coronary Heart Disease were used as covariates because of their association with CVD risk and included: age, sex at birth, race/ethnicity, total cholesterol, HDL, SBP, diabetes diagnosis, any use of blood pressure medications, and self-reported smoking.

### Analyses

Baseline characteristics were compared descriptively by HIV status. The effect of HIV status on MI risk was assessed separately in two calendar eras using stepwise Cox proportional hazards models adjusted for demographics and Framingham risk score components. The overall sample was first examined, then stratified by cohort. Kaplan-Meier curves showing the MI incidence by calendar era were generated. Several sensitivity analyses were performed to evaluate (1) events over a 10-year period; (2) stratification by sex; and (3) alternative calendar era strata (e.g., 2005–2009; 2010–2014; 2015–2017). Alternative calendar eras were examined in KPNC only due to cohort size and availability of data. All analyses were performed using SAS, version 9.4 (Cary, NC).

## Results

### Sociodemographic and clinical characteristics at baseline

The study included 9,401 people with HIV (4,280 [3,584 KP and 696 MGB] from 2005–2009 and 5,121 [4,615 KP and 506 MGB] from 2010–2017) and 29,418 matched people without HIV (14,059 [10,740 KP and 3,319 MGB] from 2005–2009 and 15,359 [13,857 KP and 1,502 MGB] from 2010–2017) overall. People with HIV and people without HIV were well-matched by age, sex, race/ethnicity, and CVD risk factors in each calendar era (Table 1). Median follow up time was 4.78 (IQR = 2.24, 5.0) and 4.93 (IQR = 2.08, 5.00) years for people with HIV and people without HIV, respectively, overall and by calendar era (2005–2009: 5.00 [2.60, 5.00] vs. 5.00 [2.30, 5.00] and 2010–2017: 3.95 [1.98, 5.00] vs. 4.09 [1.90, 5.00]). Overall percentage lost to follow up or death was comparable by HIV status overall (44.0% for people with HIV and 43.0% for people without HIV, respectively) and by calendar era (38% vs. 39.0% for 2005–2009 and 49.0% vs. 47.5% for 2010–2017).

**Table 1:** Overall cohort baseline characteristics.

	People with HIV		People without HIV	
**Baseline Calendar Era**	**2005-2009**	**2010-2017**	**2005-2009**	**2010-2017**
N	4,280	5,121	14,059	15,359
Mean age, years, n (SD)	44.5 (9.3)	43.7 (11.4)	44.2 (11.9)	43.3 (12.9)
Men, % (n)	87 (3714)	89.3 (4574)	85 (11946)	89.6 (13762)
Race, % (n)				
White	53.3 (2265)	48.6 (2483)	50.7 (7085)	49.4 (7572)
Black	16.5 (703)	17.8 (907)	19.1 (2664)	16.9 (2584)
Other	30.2 (1283)	33.6 (1719)	30.2 (4212)	33.7 (5158)
Mean total cholesterol, mg/dL, n (SD)	182.6 (45.1)	177.2 (40.9)	182.9 (40.8)	178.4 (39.4)
Mean HDL cholesterol, mg/dL, n (SD)	42.6 (13.6)	45.8 (13.8)	43.7 (12.0)	45.4 (12.2)
Mean systolic blood pressure, mmHg, n (SD)	123.0 (15.5)	123.6 (14.5)	123.8 (15.0)	123.0 (14.8)
Current smoker, % (n)	26.6 (1139)	22 (1125)	28.0 (3942)	23 (3529)
Hypertension medications, % (n)	25.8 (1103)	24.9 (1276)	28.1 (3947)	23.2 (3564)
Diabetes, % (n)	5.9 (252)	4.7 (243)	6.6 (930)	5.5 (843)
*HIV-Specific*				
HIV RNA > 400 copies/mL, % (n)	39 (1642)	23 (1175)		
Mean CD4, cells/µL (SD)	470 (261.8)	587 (297.7)		
ART use, % (n)	76 (3244)	88 (4505)		
Mean years HIV (SD)	7.8 (6.8)	9.1 (8.7)		
Prior ART experience (among ART users), %, (n)				
NNRTI	52 (2226)	46 (2356)		
PI	54 (2311)	27 (1383)		
INSTI	3 (128)	40 (2048)		

As shown in the table, CVD risk issues were comparable between people with HIV and people without HIV in calendar era 2005–2009. During calendar era 2010–2017, both people with HIV and people without HIV had higher baseline levels of mean HDL, while mean total cholesterol, mean SBP, use of hypertension medications, and smoking status all were lower in comparison with 2005–2009. Among people with HIV, comparing 2005–2009 with 2010–2017, a decreased percentage of participants with baseline HIV RNA > 400 copies/mL and increased Mean CD4, ART use, and years since HIV diagnoses, respectively were observed. Differences were identified in prior ART class experience, with a greater prevalence of integrase inhibitor use in the more recent calendar era.

### Cumulative Incidence of MI by HIV Status

In 2005–2009, KPNC contributed 138 MI events (33 people with HIV and 105 people without HIV) and MGB contributed 14 MI events (2 people with HIV and 12 people without HIV). The MI rate (number of events accrued per group per 100 person-years) was comparable between people with HIV and people without HIV overall (0.21 vs. 0.22) as well as at KPNC (0.24 vs. 0.25) and at MGB (0.08 vs. 0.10). In 2010–2017, KPNC contributed 120 MI events (39 people with HIV and 81 people without HIV), while MGB contributed 10 MI events (4 people with HIV and 6 people without HIV). The MI rate was higher in people with HIV than people without HIV overall (0.25 vs. 0.16), at KPNC (0.25 vs. 0.17) and at MGB (0.22 vs. 0.12).

**Fig 1:**
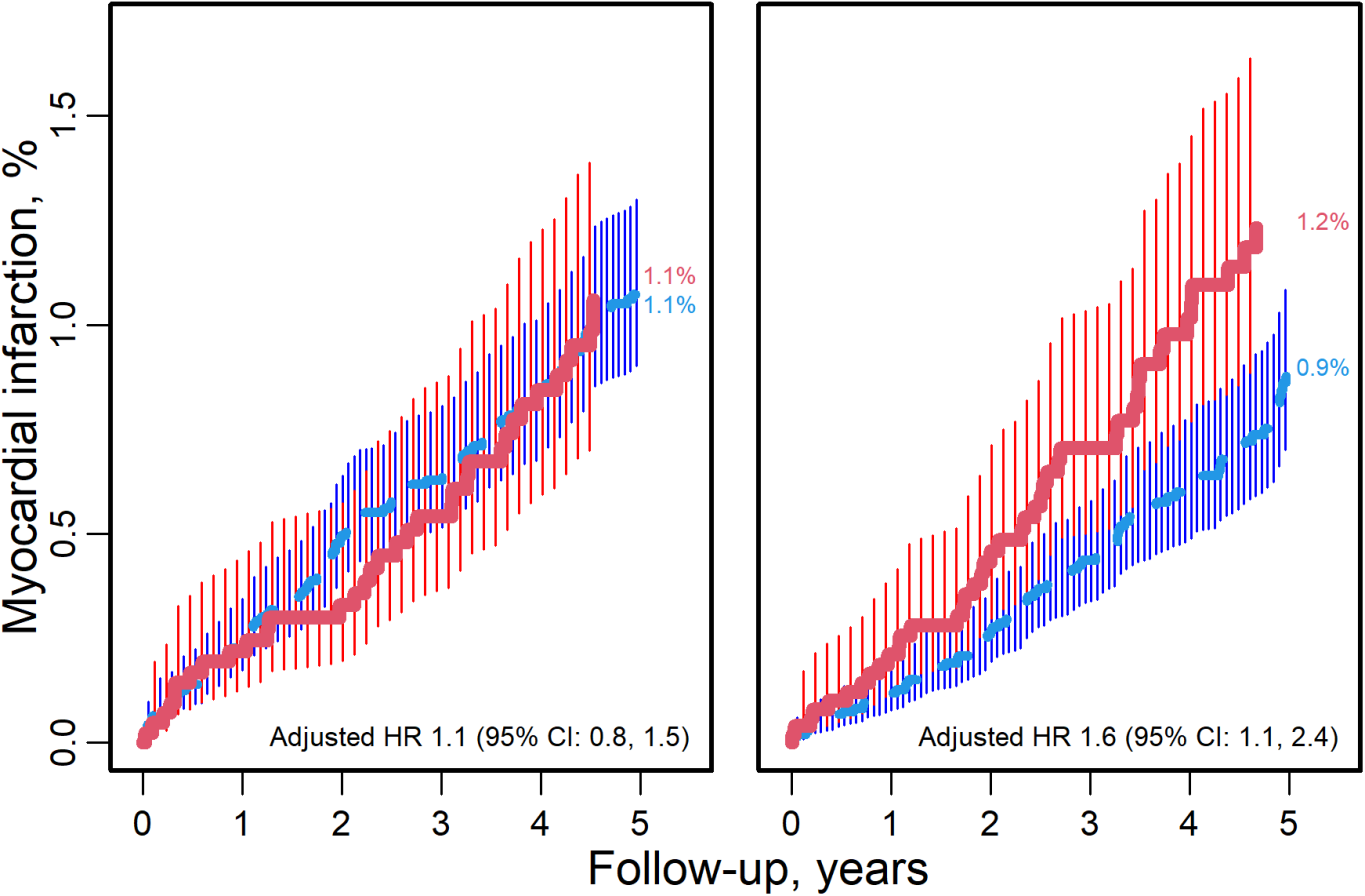
5-year cumulative incidence rates of myocardial infarction. Kaplan Meier curves illustrating the cumulative incidence rates of MI over 5 years from 2005 to 2020, presented by HIV status, with people with HIV represented in red and people without HIV in blue. Hazard ratios adjusted for age and sex are shown in the table immediately below the curves.

The rates of MI over 5 years were similar by HIV status in 2005–2009 but higher for people with HIV compared with people without HIV in 2010–2017 (Fig 1). Between 2005–2009, no difference was found in MI rates by HIV status with 1.1% risk of MI over 5 years for both people with HIV and people without HIV (P = 0.83). Between 2010–2017, the risk over 5 years was 1.2% for people with HIV and 0.9% for people without HIV (P = 0.03).

Adjusted hazard ratios (aHR) for MI by HIV status, using people without HIV as a reference, showed no difference in risk overall (1.10 [0.76, 1.60]; P = 0.61]; in KPNC (1.03 [0.69, 1.51]; P = 0.90) or MGB (1.20 [0.25, 5.78]; P = 0.82) during calendar era 2005–2009. There was > 60% higher risk of MI for people with HIV overall (1.66 [1.15, 2.39]; P = 0.007), in KPNC (1.62 [1.11, 2.39]; P = 0.014), and in MGB (2.06 [0.56, 7.50]; P = 0.28) during calendar era 2010–2017 compared with people without HIV. Interactions between calendar eras and HIV status were not significant (P-interaction = 0.12).

### Sensitivity analyses

Sensitivity analyses to evaluate events over 10 years showed similar overall results by HIV status. Significant differences were observed in calendar era 2010–2017 for adjusted models (1.59 [1.17, 2.16]; P = 0.0028), but not 2005–2009 (1.20 [0.92, 1.57]; P = 0.18). Interactions between calendar era and HIV status were not significant (P-interaction = 0.19)

Stratifying by sex also showed similar results between calendar eras with reduced precision for women. No difference was found in risk in calendar era 2005–2009 for men (aHR 1.12 [0.76, 1.65]; P = 0.58) or women (0.76 [0.17, 3.49]; P = 0.72). Differences were observed in calendar era 2010–2017 for men (1.53 [1.05, 2.23]; P = 0.03) and women (4.71 [0.64, 34.46]; P = 0.13). Interactions between HIV status and calendar era were not significant for men (P-interaction = 0.71) or women (P-interaction = 0.59).

Alternative era strata were also evaluated in KPNC only, with similar inferences in adjusted models for 2005–2009 (1.03 [0.70, 1.51] P = 0.90), 2010–2014 (1.68 [1.05, 2.69] P = 0.030), and 2015–2020 (1.38 [0.69, 2.77] P = 0.37). Interaction between HIV status and calendar eras was not significant (P-interaction = 0.27)

## Discussion

Among people with HIV and people without HIV with similar CVD risk profiles at baseline, we observed a 60% higher risk in people with HIV comparing 2010–2017 with 2005–2009. These results appear to be driven by a decrease in MI risk for people without HIV, which was not evident for people with HIV.

Previous results in population-based cohorts considering CVD risk in people with HIV during similar time periods have been mixed. A similar study in France during the early calendar era found people with HIV to have a significantly higher risk of MI compared with people without HIV [23], while Masiá and colleagues found a decreasing incidence of MI in people with HIV in Spain despite an overall higher CVD incidence in people with HIV compared to people without HIV [24]. Previous data from KPNC showed a narrowing of the gap between MI-incidence rate ratios by HIV status from 2004 to 2011 [25], and our current study extended that follow up period by 9 years. The declining risk found in recent years is consistent with our previous finding of no difference in the earlier calendar period. However, another population-based matched cohort in the U.K, found no difference in MI incidence in people with HIV versus people without HIV (1.30 [95% CI, 0.94–1.79]), with no difference by HIV status for 2000–2009 or 2010–2019 [26]. Our findings of stable MI rates among people with HIV in an era of exposure to broadly disseminated CVD risk reduction efforts, point to the need for targeted development and distribution of prevention efforts for people with HIV.

HIV-specific factors, such as chronic inflammation, concomitant infection with other viruses, and possible cardiometabolic effects of ART, may have prevented people with HIV from realizing the same improvements in MI risk as people without HIV. Specific to the cardiometabolic effects of ART, the calendar periods evaluated in this analysis reflect periods of changing patterns of medication usage, such as abacavir, which has conflicting evidence linking its use to an increased risk of MI. While data from several individual studies, including the D:A:D study [27], have found an increased relative risk for people with HIV with exposure to abacavir versus not, meta-analyses on clinical trial data by GlaxoSmithKline [28], the US Food and Drug Administration [29], and other researchers have not found the same results [30]. Assuming there is an association between abacavir and MI, risk of MI would be expected to decrease over time with decreasing abacavir use, and the opposite was observed in the current study.

In another example of the influence of prescription drug use, statins have been shown to lower risk of MI and other major adverse cardiovascular events (MACE) in people with HIV in the recent REPRIEVE trial [31]. These findings among people with HIV who would not have otherwise met current guideline criteria in which statin initiation is currently recommended suggest a potential role for non-traditional, HIV-associated factors not addressed by general population prevention guidelines. In February 2024, The Department of Health and Human Services Guidelines Panel for the Use of Antiretroviral Agents in Adults and Adolescents with HIV released recommendations for statin therapy in people with HIV [32]. The data presented here supports the need for additional studies to determine if implementation of these new guidelines will result in a reduction in CVD disparities for people with HIV.

A continued emphasis on immediate and continuous ART is essential for not only HIV control, but also for reduction in inflammation and cardiovascular risk [13,33]. Previous work in our setting showed an increased risk of MI among people with HIV with low CD4 counts compared to people with HIV with recent or nadir CD4 ≥ 500 cells per microliter compared to people without HIV [13]. Accurate prediction of CVD risk for people with HIV also remains an important tool in reducing morbidity and mortality in people with HIV, but continues to be a challenge for clinicians as current prediction models have been shown to underestimate risk, particularly for low- to moderate-risk individuals, who may benefit most from early clinical interventions [34].

A major advantage of the study was the large cohort of people with HIV and a well-matched comparison of people without HIV with similar CVD risk. Data reflected a high-quality ascertainment of HIV status and MI events and similar follow-up for MI by calendar era (up to 5 years). Results were consistent in two large, geographically distinct US-based HIV cohorts, which suggests a true lack of improvement for people with HIV compared with people without HIV. However, this study had some limitations. First, analyses were based on information collected during routine healthcare encounters and therefore may not include some lifestyle and behavioral factors with implications for CVD risk. This also resulted in our inability to assess the potential impact of specific social inequalities such as income and education level on rates of MI. However, such differences are likely minimized among insured cohorts, and it is unlikely that differences in social inequalities by HIV status changed over time. Perceived CVD risk may have influenced the timing of CVD preventive efforts for providers, however the increased risk of CVD for people with HIV was well established during the study period and CVD prevention guidelines specific to people with HIV were consistent during the entire study period, which likely minimized any potential selection bias. Second, our population was largely male and results may have limited generalizability to women. The study also predated the REPRIEVE trial findings and did not specifically evaluate differences in prescription or uptake of statins or other cardiovascular-related medications, which may modify MI risk, however, lab values that would be impacted by their use were included in analysis. The study also had a 5-year follow up period, which is short relative to CVD risk, but we evaluated 10-year outcomes in sensitivity analyses with similar results. Additionally, we did not evaluate the potential impact of competing risks, but note that the percentage of individuals lost to follow up or death was comparable by HIV status in each calendar era, suggesting the potential impact on our relative effect measures would be minimal. Finally, we were unable to distinguish MI type I vs. type II, as done in previous studies [35]. This is a potential limitation, given that previous work by Crane et. al. found MI Type II accounted for half of all MIs observed in people with HIV, with significant differences by age, while recent estimations of MI in a more general US population found that only 25% were MI Type II [36]. However, this analysis was focused on changes in rates by HIV status over time and it is unlikely that distribution of MI type would have changed significantly.

Future studies should continue to monitor trends in CVD risk disparities between people with HIV and people without HIV, explore disparities by MI type and the clinical implications that result, as well as optimize management of CVD risk in an aging HIV population, including exploring the impact of exposure to specific ART, such as abacavir, or INSTI vs. non-INSTI, over time. Trends should be reassessed after the implementation of new HIV-directed guidelines for statin use to evaluate their effect on MI rates in people with HIV. Additionally, focusing on the inclusion of HIV-specific risk factors into CVD risk prediction models and monitoring of risks associated with HIV-associated inflammation may reduce disparities in risk with the general population.
